# Supplementary material for: The Aerobic Denitrification Characteristics of a Halophilic Marinobacter sp. Strain and Its Application in a Full-Scale Fly Ash-Washing Wastewater Treatment Plant
Source: Microorganisms. 2025 May 30;13(6):1274. doi: 10.3390/microorganisms13061274 (PMC12194979; doi:10.3390/microorganisms13061274)
Supplement: Supplementary file 1 [file microorganisms-13-01274-s001.zip › microorganisms-3601875-supplementary.pdf]

Supplementary materials for Aerobic Denitrification Characteristics of a halophilic Marinobacter sp. Strain and Its Application in a Full-Scale Fly Ash Washing Wastewater Treatment Plant

Mengyang Guo [1,2], Kai Liu [1,2], Hongfei Wang [1,2], Yilin Song [1,2], Yingying Li [1,2], Weijin Zhang [1,2], Jian Gao [1,2], Mingjun Liao [1,2*]

[1]Hubei Key Laboratory of Environmental Geotechnology and Ecological Remediation for Lake & River, Hubei University of Technology, China,

[2]Key Laboratory of Intelligent Health Perception and Ecological Restoration of Rivers and Lakes, Ministry of Education, Hubei University of Technology, Wuhan 430068, China

15327367382@163.com(M.G.); lk52598@163.com(K.L.); m18803988359@163.com(H.W); songyilin0610@163.com(Y.S.); lyy818927@163.com(Y.L.); 13361590405@163.com(W.Z.); jgao13@hotmail.com(J.G.)

* Correspondence: lmj1112@163.com

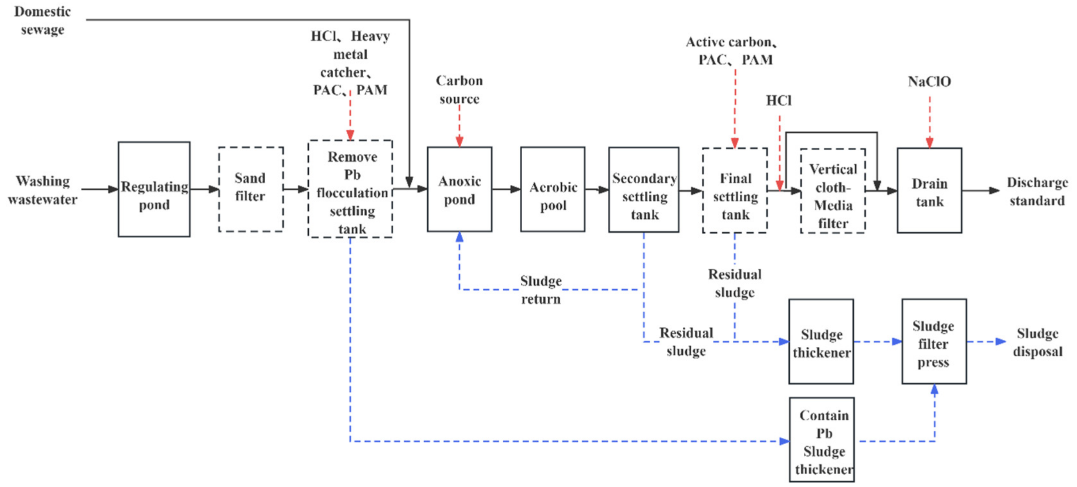

Figure S1. Process Flow Diagram for the Treatment of Wastewater from Salt-Containing Fly Ash Washing

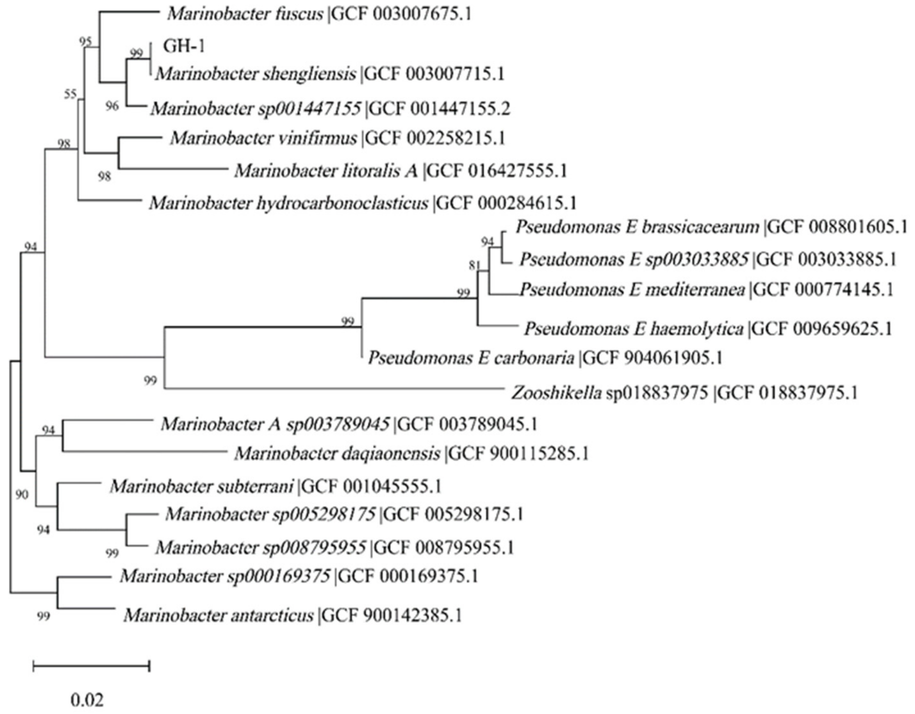

Figure S2. Phylogenetic tree of strain GH-1 constructed using the Neighbor-Joining method based on partial 16S rRNA gene sequences.

Table S1. Main Types, Composition, and Volume of Other Wastewater

Wastewater Type	Main Source	Main Components	Main Water Quality Indicators (mg/L)	Water Volume* (m^3/d)
Primarily spray wastewater	During the loading and unloading of fly ash, a spray method is used to prevent the fly ash from dispersing and causing pollution.	Water, suspended solids, A small amount of heavy metals.	Suspended Solids (SS): 200 - 2000 mg/L	1.0
Acidic gas absorption wastewater	The acidic gas scrubbing wastewater generated during the subsequent	Water, acids, dissolved salts, particulates	Chlorides (Cl^-): 100 - 500 mg/L Suspended Solids (SS):	3.5

	melting of washed fly ash.		50 - 400 mg/L<br>pH Value: 1 - 5<br>Lead (Pb): 0.1 - 25 mg/L<br>Cadmium (Cd): 0.01 - 5 mg/L	
Ground flushing wastewater	Wastewater from cleaning the floor of the fly ash washing room	Water, suspended solids, A small amount of heavy metals.	Suspended Solids (SS): 500 - 5000 mg/L<br>Lead (Pb): 0.1 – 0.5 mg/L	1.5
Cooling water	Circulating cooling water	Water<br>Hardness Ions	Calcium ions (Ca^{2+}):	1.2

	for the melting boiler.		20 - 80 mg/L<br>Magnesium ions (Mg^{2+}): 10 - 30 mg/L	
Domestic sewage within the plant area	The domestic sewage in the factory area is mainly generated from employee washing and toilet flushing.	water, organic matter, nutrients, pathogens, suspended solids	Biochemical Oxygen Demand, BOD_5： 100 - 400 mg/L<br>Ammonia Nitrogen, NH_3-N： 20 - 100 mg/L<br>Total Phosphorus, TP： 5 - 20 mg/L	2.5

		Total Nitrogen, TN：30 - 150 mg/L<br><br>pH: 6.5 - 8.5

*The aforementioned wastewater has undergone pretreatment (e.g., acid-base neutralization, sedimentation, etc.) before being mixed.

Table S2. Part of halophilic gene proteins of strain GH-1

TGene ID	NR Hit	NR Description	Gene Len (bp)	Hit Len (bp)	Identity (%)	Coverage (%)	Evalue
gene3500	WP_152439199.1	MULTISPECIES: cyclopropane-fatty-acyl-phospholipid synthase family protein	1314	437	99.1	99.77	0
gene 3500	WP_072676191.1	cyclopropane-fatty-acyl-phospholipid synthase family protein	1314	437	99.1	99.77	0
gene 3500	BBJ05667.1	cyclopropane-fatty-acyl-phospholipid synthase	1314	436	99.1	99.54	0
gene 0050	MAO15116.1	sodium:proton antiporter	1323	440	99.5	99.77	2.24E-288
gene 0050	WP_138437370.1	Na+/H+ antiporter family protein	1323	440	98.9	99.77	5.27E-287
gene 0050	WP_072675823.1	Na+/H+ antiporter family protein	1323	440	98.6	99.77	1.24E-285
gene 3962	WP_106694612.1	sodium/proton antiporter NhaB	1506	501	99.8	99.8	0
gene 3962	WP_061333197.1	sodium/proton antiporter NhaB	1506	501	99.6	99.8	0
gene 3962	WP_072675907.1	sodium/proton antiporter NhaB	1506	501	99.6	99.8	0
gene 3962	MAO11868.1	sodium/proton antiporter NhaB	1506	501	99.6	99.8	0
gene 3962	WP_138437423.1	MULTISPECIES: sodium/proton antiporter NhaB	1506	501	99	99.8	0
gene 1627	WP_138436473.1	MULTISPECIES: sodium/proline symporter PutP	1491	496	99.8	99.8	0
gene 1627	WP_230712692.1	sodium/proline symporter PutP	1491	496	99.6	99.8	0
gene 1627	WP_106695482.1	sodium/proline symporter PutP	1491	496	99.4	99.8	0
gene 1627	WP_058090422.1	sodium/proline symporter PutP	1491	496	99.4	99.8	0
gene 1627	WP_072677576.1	sodium/proline symporter PutP	1491	496	97.8	99.8	0

gene 0029	WP_061331400.1	MULTISPECIES: Trk system potassium transporter TrkA	1404	467	100	99.79	0
gene 0029	WP_058091830.1	Trk system potassium transporter TrkA	1404	467	99.8	99.79	0
gene 0029	MBE0486851.1	Trk system potassium transporter TrkA	1404	467	97.2	99.79	0
gene 0029	WP_022990431.1	Trk system potassium transporter TrkA	1404	467	94.4	99.79	0
gene 2046	WP_152438165.1	MULTISPECIES: TrkH family potassium uptake protein	1419	472	100	99.79	0
gene 2046	WP_058089830.1	TrkH family potassium uptake protein	1419	472	98.1	99.79	0
gene 2046	WP_061332927.1	TrkH family potassium uptake protein	1419	472	97.5	99.79	0
gene 2046	WP_106695771.1	TrkH family potassium uptake protein	1419	472	97.7	99.79	0
gene 2046	WP_227513237.1	TrkH family potassium uptake protein	1419	464	98.1	98.09	0
gene 2053	PPK52603.1	trk system potassium uptake protein TrkH	1368	455	93	99.78	7.18E-288
gene 2054	WP_152438172.1	MULTISPECIES: TrkA family potassium uptake protein	654	217	100	99.54	2.57E-150
gene 2054	WP_106695779.1	TrkA family potassium uptake protein	654	217	97.7	99.54	4.09E-147
gene 2054	WP_023010785.1	TrkA family potassium uptake protein	654	217	96.3	99.54	9.64E-146
gene 2054	WP_022992340.1	MULTISPECIES: TrkA family potassium uptake protein	654	217	96.3	99.54	2.76E-145
gene 2055	WP_228715526.1	MULTISPECIES: TrkH family potassium uptake protein	1461	486	100	99.79	0
gene 2055	WP_244178320.1	TrkH family potassium uptake protein	1461	486	98.6	99.79	0
gene 2055	PSF11758.1	potassium transporter TrkH	1461	462	98.5	94.86	0
gene 2055	WP_230707007.1	TrkH family potassium uptake protein	1461	483	94.8	99.18	0
gene 2055	WP_023009734.1	TrkH family potassium uptake protein	1461	486	92.6	99.79	1.87E-310
gene 2049	WP_152438168.1	MULTISPECIES: sensor histidine kinase KdpD	2679	892	100	99.89	0

gene 2049	WP_106695774.1	sensor histidine kinase KdpD	2679	892	97.6	99.89	0
gene 2050	WP_228715525.1	MULTISPECIES: potassium-transporting ATPase subunit KdpC	606	201	99.5	99.5	8.64E-132
gene 2050	WP_022992334.1	MULTISPECIES: potassium-transporting ATPase subunit KdpC	606	201	95.5	99.5	1.54E-125
gene 2050	WP_023010779.1	potassium-transporting ATPase subunit KdpC	606	201	93.5	99.5	4.22E-123
gene 2050	WP_228743670.1	potassium-transporting ATPase subunit KdpC	606	201	94	99.5	2.44E-122
gene 2050	MBW3225399.1	potassium-transporting ATPase subunit KdpC	606	200	94	99	1.36E-121
gene 2051	WP_216646181.1	MULTISPECIES: potassium-transporting ATPase subunit KdpB	2019	665	100	98.81	0
gene 2052	WP_152438169.1	MULTISPECIES: potassium-transporting ATPase subunit KdpA	1713	570	100	99.82	0
gene 2052	WP_104415515.1	potassium-transporting ATPase subunit KdpA	1713	570	97.2	99.82	0
gene 2052	WP_022992336.1	MULTISPECIES: potassium-transporting ATPase subunit KdpA	1713	570	97.2	99.82	0
gene 2052	MAI34162.1	potassium-transporting ATPase subunit KdpA	1713	570	97	99.82	0
gene 3882	MAO13335.1	betaine-aldehyde dehydrogenase	1470	489	98.6	99.8	0
gene 3882	WP_152439474.1	MULTISPECIES: betaine-aldehyde dehydrogenase	1470	489	98.4	99.8	0
gene 3882	WP_106693245.1	betaine-aldehyde dehydrogenase	1470	489	98.6	99.8	0
gene 3882	WP_058090851.1	betaine-aldehyde dehydrogenase	1470	489	98.2	99.8	0
gene 3882	BBJ06044.1	NAD/NADP-dependent betaine aldehyde dehydrogenase	1470	489	98	99.8	0
gene 3987	WP_106694595.1	ectoine hydroxylase	936	311	100	99.68	1.14E-234
gene 3987	WP_058090918.1	ectoine hydroxylase	936	311	98.4	99.68	3.65E-231
gene 3987	TVT33926.1	ectoine hydroxylase	936	311	90	99.68	2.50E-213
gene 3987	WP_023008494.1	ectoine hydroxylase	936	311	89.4	99.68	7.18E-213

gene 0078	WP_008176749.1	MULTISPECIES: ectoine synthase	396	131	100	99.24	6.72E-95
gene 0078	PTB91498.1	L-ectoine synthase	396	131	99.2	99.24	1.93E-94
gene 0078	WP_247844098.1	ectoine synthase	396	131	99.2	99.24	1.93E-94
gene 0078	WP_058092911.1	MULTISPECIES: ectoine synthase	396	131	99.2	99.24	2.74E-94
gene 0078	WP_152436973.1	MULTISPECIES: ectoine synthase	396	131	99.2	99.24	2.74E-94
gene 2556	WP_152438574.1	MULTISPECIES: alkaline phosphatase D family protein	1788	595	97	99.83	0
gene 2556	WP_106695114.1	alkaline phosphatase D family protein	1788	595	97	99.83	0
gene 2556	WP_106671608.1	alkaline phosphatase D family protein	1788	595	89.2	99.83	0
gene 2556	BBJ04612.1	alkaline phosphatase	1788	547	96	91.76	0
gene 3503	MAO13172.1	alkaline phosphatase	1902	633	95.7	99.84	0
gene 3503	BBJ05670.1	alkaline phosphatase	1902	633	94.6	99.84	0
gene 3632	MAO11877.1	alkaline phosphatase	1764	580	99.3	98.64	0
gene 3632	WP_106693999.1	alkaline phosphatase D family protein	1764	580	98.8	98.64	0
gene 3632	BBJ05785.1	alkaline phosphatase	1764	580	98.3	98.64	0
gene 3632	WP_152439281.1	MULTISPECIES: alkaline phosphatase D family protein	1764	580	98.3	98.64	0
gene 0729	MAO12390.1	malate dehydrogenase	1266	421	99.8	99.76	7.40E-298
gene 0729	WP_061332389.1	MULTISPECIES: malate dehydrogenase	1266	421	99.5	99.76	3.01E-297
gene 0729	WP_138438144.1	malate dehydrogenase	1266	421	99.3	99.76	1.23E-296
gene 0729	WP_072678332.1	malate dehydrogenase	1266	421	99	99.76	4.99E-296
gene 0729	WP_058090080.1	malate dehydrogenase	1266	421	98.3	99.76	4.78E-294

Gene ID: The identification number of the gene; NR Hit: The name of the target sequence matched in the NR database; NR Description: Functional description of the gene; Gene Len (bp): Length of the gene; Hit Len (bp): Length of the matched gene; Identity (%): Sequence identity of the match; Coverage (%): Coverage of the matched sequence; Evalue: Evaluation of the reliability of the Score value, the smaller the value, the more reliable it is.
